# Guar Gum as an Eco-Friendly Corrosion Inhibitor for Pure Aluminium in 1-M HCl Solution

**DOI:** 10.3390/ma12162620

**Published:** 2019-08-16

**Authors:** Gaetano Palumbo, Katarzyna Berent, Edyta Proniewicz, Jacek Banaś

**Affiliations:** 1 Faculty of Foundry Engineering, AGH University of Science and Technology, Reymonta St. 23, 30-059 Krakow, Poland; 2 Academic Centre for Materials and Nanotechnology, AGH University of Science and Technology, Mickiewicza St. 30, 30-049 Kraków, Poland

**Keywords:** corrosion inhibition, pure aluminium, guar gum, natural inhibitor, FTIR, Raman

## Abstract

Guar gum (GG) was investigated as a possible eco-friendly corrosion inhibitor for pure aluminium in a 1-M HCl solution at different temperatures and immersion times using gravimetric and electrochemical techniques. The results showed that GG was a good corrosion inhibitor for pure aluminium in the studied environment. The inhibition efficiency of GG increased with increasing inhibitor concentration and immersion time but decreased with increasing temperature. Polarisation measurements revealed that GG was a mixed type inhibitor with a higher influence on the cathodic reaction. The adsorption behaviour of the investigated inhibitor was found to obey the Temkin adsorption isotherm and the calculated values of the standard free adsorption energy indicate mixed-type adsorption, with the physical adsorption being more dominant. The associated activation energy (*E_a_*) and the heat of adsorption (*Q_a_*) supported the physical adsorption nature of the inhibitor. Fourier-transform infrared spectroscopy (FTIR) and Raman/SERS were used to explain the adsorption interaction between the inhibitor with the surface of the metal. The results suggested that most inhibition action of GG is due to its adsorption of the metal surface via H-bond formation.

## 1. Introduction

Aluminium is one of the most important and the second most-used metal in the world. Aluminium has low density, excellent thermal and electric conductivity, an attractive appearance, and good corrosion resistance. Due to these unique properties, aluminium is commonly used in many industries. The good corrosion resistance of aluminium is due to the formation of a thin inert oxide layer on top of its surface upon exposure to the atmosphere. However, this protective layer is easily damaged when exposed to aggressive solutions, which in turn lead to corrosion of the metal. Strong acids such as hydrochloric and sulphuric acids are often used in the aluminium industry for pickling and for chemical or electrochemical etching of its surface. One practical and cost-effective method to mitigate the aluminium corrosion in such aggressive environments is the use of corrosion inhibitors. The corrosion inhibition effect of these compounds occurs via adsorption of their molecules on the metal surface, thus isolating the metal surface from the corrosive agents. Inorganic inhibitors such as chromates, dichromate, phosphates, etc. have been found to exhibit excellent anticorrosion properties. However, their toxicity to both environment and human health led the Environment European Commission (EEC) to limit their use (i.e., Directive 76/464/EEC). Their limitation has brought many researchers to look forward towards the use of alternative and more eco-friendly corrosion inhibitors. Plants extract products [1,2,3] have been highly considered as eco-friendly corrosion inhibitors. They are abundant and environmentally sustainable and have appreciable solubility. Recently, naturally occurring polysaccharides and their derivatives have also been found to be excellent alternative corrosion inhibitors. Unlike small molecule, polymers can cover larger surface areas on the metal surface due to their multiple adsorption sites for bonding with the metal surface. Therefore, they are expected to be better corrosion inhibitors than their monomer analogues [4]. The use of the natural polymer gum arabic has been reported to inhibit the corrosion of mild steel and aluminium in H_2_SO_4_ solutions [5,6,7] and aluminium in NaOH solutions [8,9]. The use of polysaccharides such as xanthan gum [10] and plantago [11] to improve the corrosion resistance of mild steel in HCl solutions has been reported as well. All these studies reported that the corrosion inhibition action of these natural polymers was attributed to their larger molecular size and to the presence of heteroatoms with unshared electron pairs such as O, S, and N [1,2,3,4,5]. These electron-rich heteroatoms work as adsorptive centres, allowing the inhibitor to be adsorbed on the surface of the metal and, thus, hindering the corrosion of the metal.

**Figure 1:**
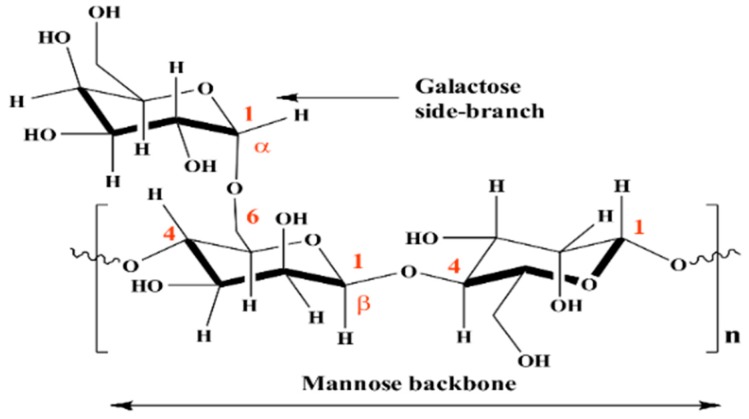
Chemical structure of Guar gum (GG).

Guar gum (GG) is a natural polymer extracted from the seed of the Guar plant *Cyamopsis tetragonolobus* of the Leguminosae family [12]. GG is a high-molecular-weight polysaccharide consisting of a backbone of D-mannopyranose (M) monomer units attached to each other by β-(1→4) linkage with a side-branch consisting of a single α-D-galactopyranose (G) linked to the mannose unit by α-(1→6) linkages (Figure 1) [12]. GG is commonly used in the food, pharmaceutical, and cosmetics industries, mostly due to its low cost and its specific properties, such as binding agent, stabiliser, and thickening agent [12]. However, as witnessed in an increasing number of publications, GG is also emerging as a promising candidate as a corrosion inhibitor of metal infrastructures [13,14,15,16,17,18]. Moreover, GG being a biodegradable, nontoxic natural compound provides more environmental benefits than the common toxic inorganic inhibitors, with great benefits for the environment. In 2004, Abdallah [13] reported the first-ever attempt involving the use of GG as a corrosion inhibitor of carbon steel in a H_2_SO_4_ solution. Abdallah reported that GG provided good corrosion protection for the metal surface, with the maximum value of the corrosion efficiency found to be 93.88%. The author believed that the presence of oxygen atoms in the repeating mannose units of the GG structure made possible its adsorption on the metal surface. After this first report, several studies have been published on the use of GG as a corrosion inhibitor in acid and neutral solutions [14,15,16,17]. All these studies reported that GG was an effective corrosion inhibitor. 

Due to the encouraging results presented by these studies and the continuous research of affordable and eco-friendly corrosion inhibitors, this research aimed to investigate, for the first time, the effectiveness of Guar gum as a corrosion inhibitor for pure aluminium in a 1-M HCl solution. To this end, the anticorrosive properties of Guar gum were studied by gravimetric and electrochemical techniques such as electrochemical impedance spectroscopy and potentiodynamic polarisation curves. Scanning electron microscopy (SEM), Fourier-transform infrared spectroscopy (FTIR), and Raman/SERS spectroscopy were also employed to characterize the surface of the metal in order to support the electrochemical results. 

## 2. Experimental Procedures

### 2.1. Material and Solution

The study was carried out on pure aluminium (99.99%). The specimens were embedded in a PTFE cylinder block, with the flat surface area (e.g., 0.52 cm^2^) exposed to the solution. Each time prior to a test, the exposed surface was ground with silicon carbide abrasive paper up to 1200 grit. The surface was finished with levigated alumina, then cleaned ultrasonically in deionized water, and dried by absolute ethanol just before immersion in the solution. Each experiment was performed with a newly polished electrode. The experiments were carried out in a 1-M HCl solution prepared from analytical reagent grade hydrochloric acid and pure, deionized water with an electrical resistivity of 0.055 µS/cm at T = 25 °C. Guar gum was purchased from Sigma-Aldrich (Warsaw, Poland) and used without further purification. GG was added to the blank solution to obtain the desired concentrations (i.e., from 0.1 g/L up to 0.4 g/L).

### 2.2. Gravimetric Measurement

The gravimetric experiments were performed suspending the coupons in an unstirred solution (150 ml) in the absence and presence of different concentrations of inhibitor (i.e., 0.1 up to 0.4 g/L) at 25 and 45 °C. The weight loss was determined by retrieving the coupons after 24 h of immersion by means of an analytical balance. The weight loss was calculated as the difference between the initial weight prior and weight after the immersion. The corrosion rate (*CR*) was calculated using Equation (1) [5,6,7,19]:

(1) $$CR\;\left(mg\;cm^{-2}d^{-1}\right)=\frac{\Delta W}{At}$$

where Δ*W* is the weight loss (mg), A is the area of the specimen (cm^2^), and t is the exposure time (day). The inhibition efficiency (*IE*%) was evaluated using Equation (2) [5,6,7,19]:

(2) $$IE\%=\frac{CR-CR^{\mathrm{inh}}}{CR}\times 100$$

where *CR^inh^* and *CR* are the corrosion rates of the metal with and without the addition of the inhibitor, respectively.

### 2.3. Electrochemical Measurements

The electrochemical experiments were performed in a three-electrode glass cell using a Gamry reference 600 (Gamry Instruments, Warminster, PA, USA) potentiostat/galvanostat electrochemical system. A platinum foil was used as a counter electrode (CE), and a saturated calomel electrode (SCE) was used as a reference electrode. 

Potentiodynamic measurements were carried out in the potential range from −1.2 V to −0.8 V with a scan rate of 1 mV s^−1^ after holding the specimen for 24 h at 25 and 45 °C. The corrosion current density (*I*_corr_) and the anodic (*β*_a_) and cathodic (*β*_c_) Tafel constants were determined by means of Echem Analyst 5.21 software. *IE*% was calculated from the measured *I_corr_* values using Equation (3) [20]:

(3) $$IE\%=\frac{I_{corr}-I_{corr}^{inh}}{I_{corr}}\times 100$$

where *I_corr_* and *I_corr_^inh^* represent corrosion current values without and with an inhibitor, respectively. Electrochemical impedance spectroscopy (EIS) was used to study the effect of time. Each experiment was performed in unstirred solutions with a freshly prepared solution at a temperature of 25 °C. EIS tests were recorded at corrosion potentials over a frequency range of 10 kHz to 10 mHz and an amplitude of 10 mV at prefixed immersion times (i.e., 6, 12, 18, and 24 h) without and with different concentrations of the inhibitor. The EIS plots were then simulated with the help of ZSimpWin 3.5 using the appropriate equivalent circuit. *IE*% was calculated from the charge transfer resistance (*R_ct_*) obtained from the analyses process using Equation (4) [20]:

(4) $$IE\%=\frac{R_{ct}^{inh}-R_{ct}}{R_{ct}^{inh}}\times 100$$

where *R_ct_^inh^* and *R_ct_* are the charge transfer resistance values in the presence and absence of the inhibitor, respectively. Each test was run in triplicate to verify the reproducibility of the results.

### 2.4. Surface Analysis

#### 2.4.1. Fourier-Transform Infrared (FTIR) Spectroscopy

FTIR measurements were carried out using a Thermo Scientific (Indianapolis, IN, USA) Nicolet 6700 spectrophotometer equipped with an attenuated total reflectance (ATR) accessory (Indianapolis, IN, USA). The FTIR spectra of the inhibitor and the surface sample, exposed for 24 h in the presence of the optimal concentration of the inhibitor (e.g., 0.4 g/L), were recorded at a spectral resolution of 4 cm^−1^ with 128 co-added scans over the range from 3700 cm^−1^ to 700 cm^−1^. 

#### 2.4.2. Raman and SERS Measurement

The Raman and SERS (surface enhanced Raman spectroscopy) measurements were performed by using an InVia Raman Spectrometer Renishaw (Wotton-under-Edge, England) equipped with an air-cooled charge-coupled device (CCD) detector (Wotton-under-Edge, England) and a Leica microscope (50× objective) (Wotton-under-Edge, England). The spectral resolution was set at 4 cm^−1^. The 785 nm line of a diode laser was used as the excitation source. The laser power at the output was set at 10 mW (Ti plate) and 20 mW (TiO_2_NPs). Each Raman measurement was carried out with an exposure time of 40 s with four accumulations (series of 4 spectra, each accumulated 40 s = 160 s).

#### 2.4.3. Scanning Electron Microscopy (SEM)

The surface of the specimens, prepared as described above, were investigated after being exposed for 24 h in the absence and presence of the optimum concentration of the inhibitor (i.e., 0.4 g/L). After the immersion time had elapsed, the specimens were removed and rinsed with deionized water. The surface investigation was carried out using a FEI (Hillsboro, OR, USA) Versa 3D (FEG SEM) scanning electron microscope with magnification of 5000×.

## 3. Results and Discussion

### 3.1. Effect of Concentration and Temperature

#### 3.1.1. Gravimetric Measurement

Table 1 shows the corrosion rate and the corrosion inhibition efficiency values driven from weight loss measurement in a 1-M HCl solution without and with the addition of different concentrations of GG at 25 and 45 °C. The results show that, upon the addition of the inhibitor, the corrosion rate of the metal decreases and that the extent of its protection is concertation dependent. Correspondingly, the inhibition efficiency values were found to increase with the inhibitor concentration in both temperatures, with maximums at 83.19% and 60.16% at 25 and 45 °C, respectively. This behaviour is an indication that GG actually inhibited the corrosion of the metal in the acid solution. It is also clear for Table 1 that both *CR* and *IE*% are also greatly affected by system temperature. The data shows that a rise in the system temperature is followed by a decrease in inhibition efficiency. This is indicative of an inhibitor that is physically adsorbed on the aluminium surface [21,22]. However, it is worth mentioning that GG still provides good corrosion protection even at a higher temperature after 24 h of immersion (i.e., 60.16%). The high inhibition efficiency value observed may be due to the degradation of the polymer chains to smaller chains. It has been reported that solutions of GG are stable for short-term heating but degrades when heated for longer times [23]. The degradation of its polymer chains occurs through the scission of the glycosidic bonds (i.e., 1-4 *β-D* or/and 1-6 *α-D* glycosidic bonds). The result is the formation of smaller and less entangled segments which can more easily diffuse from the bulk solution to the metal surface, thus, increasing their likelihood to get adsorbed on it. Fares and coauthors also reported a similar result [24].

Table S1 (supplement material) lists the inhibition efficiency and the time of exposure at which the maximum inhibition efficiency has been calculated for various corrosion inhibitors derived from natural products. It follows from the table that these studies were mostly performed at short immersion times (i.e., up to 6 h) and that only a few of them were carried out at long immersion (i.e., 24 and 168 h). It can be seen from the table that compared to other natural corrosion inhibitors, GG can be considered a good environmentally friendly corrosion inhibitor for pure aluminium in a 1-M HCl solution for short and long exposure times and at low and high temperatures. 

#### 3.1.2. PDP Measurement

**Figure 2:**
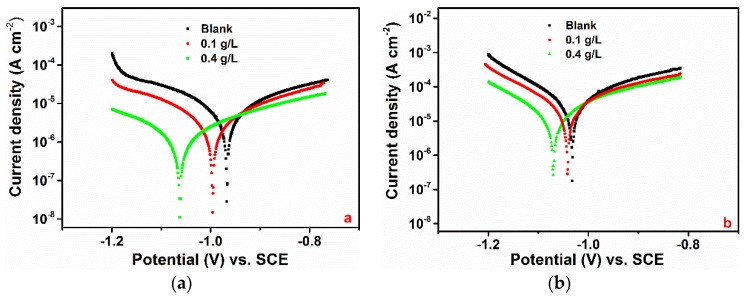
Polarisation curves for pure aluminium in 1-M HCl in the absence and presence of different concentrations of GG at 25 °C (**a**) and 45 °C (**b**) after 24 h of immersion.

Potentiodynamic polarisation curves for pure aluminium in a 1-M HCl solution in the absence and the presence of various concentrations of GG at 25 °C are shown in Figure 2a. The polarisation parameters are listed in Table 2. It can be seen from Figure 2a that *I_corr_* decreases considerably in the presence of GG and decreases with increasing inhibitor concentration. Correspondingly, the inhibition efficiency increases with the inhibitor concentration to a maximum of 84.19%. Visual inspection of the figure also reveals a marked shift in the cathodic domain of the polarisation curves toward lower current densities after the addition of GG to the blank solution. In contrast, there seems to be a small decrease in the anodic domain after the addition of GG. This behaviour implies that the rate of cathodic reaction controls the rate of corrosion. Moreover, it follows from the data presented in Table 2 that there is a gradual shift in the corrosion potential towards cathodic potential, with reference to the blank solution, as the concentration of the inhibitor increases. It is generally assumed that, if the shift in *E_corr_* after the addition of a given inhibitor is less than 85 mV, with respect to *E_corr_* of the uninhibited solution, the inhibitor can be regarded as a mixed-type inhibitor [8]. In this study, the shift in *E_corr_* after the addition of GG ranges between −29 mV to −82 mV for 0.1 and 0.4 g/L, respectively. This means that the tested inhibitor acts as a mixed-type inhibitor in agreement with the results previously reported for carbon and mild steel [3,5,8,9]. However, in the case of aluminium, GG has a higher influence on the cathodic reaction. The cathodic inhibitive action of GG can be attributed to the formation of H-bonding between the hydroxyl groups of the mannose units and the H^+^ adsorbed on the aluminium surface, thus retarding hydrogen evolution on cathodic sites of the electrode surface [14,17], as will be discussed in more detail later in Section 3.4.2. The polarisation results are in agreement with the findings observed with the gravimetric measurements reported in this study, which show that the inhibition efficiency decreases upon increasing the temperature (Table 2). Moreover, as can be seen from Figure 2b, the rise in temperature has a greater effect on the cathodic region than that observed in the anodic one. The addition of the inhibitor decreases the cathodic current density; however, its reduction is less pronounced than that observed at lower temperatures. As stated above, the inhibition of the cathodic reaction may be due to the formation of hydrogen bonds between the inhibitor and the metal surface. It is well known that the hydrogen bond is weaker than the chemical bond and that it generally weakens with increasing temperature due to larger thermal motion [25]. Therefore, the increase in temperature will enhance the metal surface kinetic energy, which has an adverse effect on the adsorption process and encourages desorption processes.

#### 3.1.3. Adsorption Study and Standard Adsorption Free Energy

**Figure 3:**
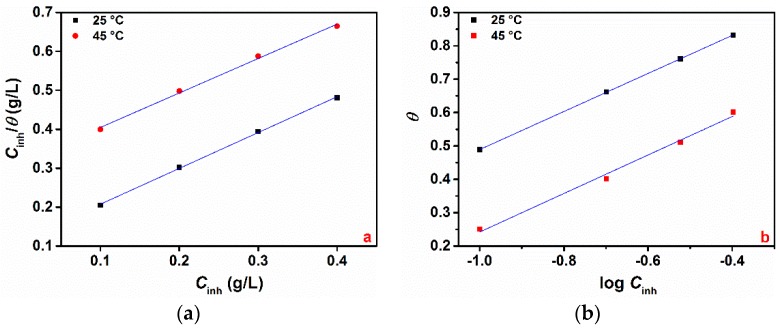
Langmuir (**a**) and Temkin (**b**) adsorption isotherms at 25 and 45 °C.

The interactions between Guar gum and the pure aluminium surface was examined by several adsorption isotherm models. Of all the adsorption isotherms tested, Langmuir (Figure 3a) and Temkin (Figure 3b) isotherm models were found to give the best description of the adsorption process for the two tested temperatures. The models considered were Equations (5) and (6):

(5) $$\mathrm{Langmuir}:\;\frac{C_{inh}}{\theta }=\frac{1}{K_{ads}}+C_{inh}$$

(6) $$\mathrm{Temkin}:\;\theta =\frac{-2.303\log K_{ads}}{2a}-\frac{-2.303\log C_{inh}}{2a}$$

where *θ* is the surface coverage, *K_ads_* the adsorption–desorption equilibrium constant, and “a” is the molecular interaction parameter. A positive value of “a” is an indication of attractive forces whereas a negative value indicates repulsive forces [14,19].

*K_ads_* is related to the free energy of adsorption (Δ*G_ads_*) by Equation (7):

(7) $$\Delta G_{ads}=-RT\;\ln\left(1\times 10^{3}K_{ads}\right)$$

where the value 1 × 10^3^ is the concentration of water molecules in solution expressed in g/L. As can been seen from Figure 3a, the plots of *C*/*θ* vs. *C* yield a straight line, with the linear correlation coefficient (R^2^) ranging between 0.996 and 0.998 (Table 3), indicating that Langmuir isotherm would be the most appropriate isotherm to describe the adsorption of GG on the metal. However, it should be pointed out that, for a given adsorption model, the goodness of the linear correlation coefficient may not offer any insight into possible adsorption mechanisms [26]. For instance, Langmuir isotherm was developed according to some specific assumptions such as the following: (i) there is no interaction between the adsorbed molecules; (ii) there is a fixed number of adsorption sites on the metal surface, and each of them holds one adsorbed species [26]. In the ideal Langmuir isotherm plot, the intercept should be zero and have a slope of unity. As can be seen from Table 3, the slope of the Langmuir plot deviated from the unity. This deviation is likely to be due to the interaction of adsorbed GG molecules on the metal surface. For this reason, the *θ* values were fitted into the Temkin adsorption model. The plot of *θ* vs. log *C* yields a straight line with the regression coefficient ranging between 0.989 and 0.999. For both isotherms, the adsorption parameters determined from the tested isotherms are presented in Table 3 and the following notes can be written:

(i) The values of Δ*G_ads_* are negative in all cases. The negative value of Δ*G_ads_* indicates that the adsorption of GG on pure aluminium in 1-M HCl is a spontaneous process [14,16,17];
(ii) The value of Δ*G_ads_* ranges between −22.48 and −21.32 kJ mol^−1^ for the Langmuir isotherm and between −27.73 and −26.92 kJ mol^−1^ for the Temkin isotherm. It should be noted that the adsorption is regarded as physisorption for values of Δ*G_ads_* up to −20 kJ mol^−1^, whereas for values more negative than −40 kJ mol^−1^, the adsorption is said to be chemisorption [5,20]. This suggests that the adsorption of GG molecules on the metal surface occurs through a mixed-type adsorption process (i.e., physical and chemical adsorption). Other authors also reported this mixed interaction adsorption process of GG in acid media [14,16,17];
(iii) The values of “a” determined from the Temkin isotherm are negative in all cases, indicating that repulsion forces exist between the adsorbed inhibitor molecules in the adsorption layer, also reported by Messali et al. [14].

#### 3.1.4. Activation Parameters

To understand the mechanism of inhibitor involved for the corrosion process of pure aluminium in 1-M HCl in the presence of Guar gum, activation parameters such as the apparent activation energy (*E_a_*) and the heat of adsorption (*Q_ads_*) were calculated. The values of the activation energy were calculated with the help of the linearized Arrhenius Equation (8):

(8) $$\log\frac{CR_{2}}{CR_{1}}=\frac{E_{a}}{2.303R}\left(\frac{1}{T_{1}}-\frac{1}{T_{2}}\right)$$

where *CR*_1_ and *CR*_2_ are the values of the corrosion rate at temperature *T*_1_ (25 °C) and *T*_2_ (45 °C), respectively. *E_a_* is the apparent activation energy; *R* is the gas constant (8.314 J mol^−1^ K^−1^). The corrosion rate (*CR*) was calculated from Equation (1), according to the ASTM Standard G 102 [27]:

The values of heat of adsorption were calculated using Equation (9) [28]:

(9) $$Q_{ads}=2.303R\left[\log\left(\frac{\theta_{2}}{1-\theta_{2}}\right)-\log\left(\frac{\theta_{1}}{1-\theta_{1}}\right)\right]\times \left(\frac{T_{1}\times T_{2}}{T_{2}-T_{1}}\right)$$

where *θ*_1_ and *θ*_2_ are values of the degree of surface coverage at temperatures *T*_1_ (25 °C) and *T*_2_ (45 °C), respectively. The activation parameters (*E_a_* and *Q_ads_*) are given in Table 4, and the following conclusions can be written:

(i) The values of *E_a_* are higher in the presence of GG than for the bank solution. It is said that higher values of *E_a_* in the presence of the inhibitor compared to the free acid solution are an indication of the inhibitive action of the inhibitor as a result of the increase in energy barrier for the corrosion process [14,29];
(ii) The values of *Q_ads_* are negative for all cases. The negative values of *Q_ads_* are an indication of the adsorption of GG on the metal surface [14,16,29];

It is generally accepted that *E_a_* values less than 40 kJ mol^−1^ are usually accepted as evidence for the mechanism of physical adsorption, whereas *E_a_* values greater than 80 kJ mol^−1^ support the chemical adsorption. Moreover, for values of Δ*H_ads_* (which is equal to *Q_ads_*) between −20 and 0 kJ mol^−1^, the process is regarded as a physisorption process, whereas values between −400 and −80 kJ mol^−1^ are regarded as a chemisorption process. It follows from the data listed in Table 4 that the values of *E_a_* and *Q_ads_* obtained in this study, ranging between 46.78 and 80.80 kJ mol^−1^ and between −46.81 and −41.45 kJ mol^−1^, respectively, further supports the proposed mixed-type adsorption nature of the process. Similar results were also reported in the literature for corrosion inhibition in the presence of Guar gum in an acid suction [14,16,17].

### 3.2. Effect of Time

**Figure 4:**
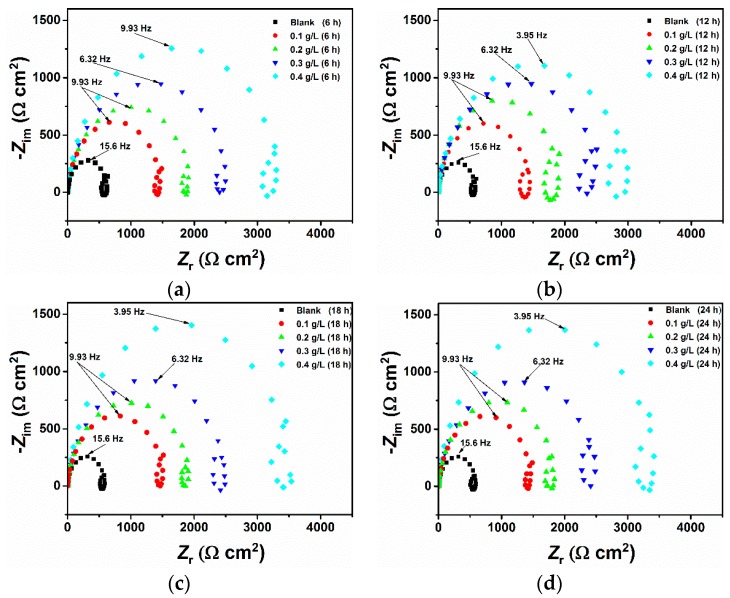
Nyquist plots recorded at open circuit potentials for pure aluminium in 1-M HCl without and with the addition of different concentrations of GG at 25 °C after 6 (**a**), 12 (**b**), 18 (**c**), and 24 (**d**) h of immersion in the absence and presence of different concentrations of the GG.

Immersion time plays an important role in assessing the stability of the inhibitive behaviour of a given corrosion inhibitor. Due to its nondestructive nature, the impedance method is well suited for long immersion time experiments and can provide valuable information about the kinetics of the electrode processes and the surface properties of the investigated systems. To this end, the effect of immersion time on the corrosion inhibition of pure aluminium in a 1-M HCl solution by Guar gum was determined using electrochemical impedance spectroscopy at 6, 12, 18, and 24 h of immersion in the absence and presence of different concentrations of the inhibitor. Figure 4 shows the Nyquist plots recorded after different immersion times with and without the presence of the inhibitor at 25 °C. Inspection of Figure 4 reveals that, in all cases, the shape of the impedance plot does not change after the addition of GG, suggesting that its addition does not alter other aspects of the corrosion mechanism. However, the figure reveals that the size of the semicircle increases with increasing concentration of the inhibitor, which is attributed to the adsorption of the inhibitor on the aluminium surface. The Nyquist plot shows that each impedance curves is characterized by three-time constants consisting of (i) a semicircle at high frequencies (HF); (ii) a small inductive loop at medium frequencies (MF); and (iii) a second capacitive semicircle at low frequencies (LF) [30,31]. Other researchers also reported similar results for the corrosion of aluminium and aluminium alloys in acid media [4,31,32,33,34]. In the literature, different explanations have been proposed on the origin of these time constants; however, there seems to be no consensus about their origin. Mansfeld et al. [35,36] suggested that the origin of the HF capacitive loop could be assigned to the relaxation process in the aluminium oxide (or hydrated oxide) film present on the aluminium surface and its dielectric properties [4] while, Brett [30] (also reported in References [33,34]) suggested that the formation of the HF capacitive semicircle may be attributed to interfacial reactions that occur at the metal/oxide/electrolyte interface. At the metal-oxide interface, aluminium is first oxidized to Al^+^ and then to Al^3+^ [33]. Thus, the high-frequency capacitive loop is related to the charge transfer of the corrosion process and double layer behaviour [37]. The origin of the inductive loop observed at MF may be attributed either to relaxation processes within the oxide film [31,38] and/or adsorption of intermediates such as H^+^ and Cl^−^ ions or inhibitor species [33]. The second capacitive loop observed at LF could be assigned to the dissolution of the layer (Al_2_O_3_) [31,37]. As also reported by other authors [37,39], due to the complex Nyquist diagrams, it is difficult to find a suitable equivalent circuit to fit the EIS results. Thus, only two important electrochemical parameters, namely the charge transfer resistance (*R_ct_*) and double layer capacitance (*C_dl_*), are calculated and listed in Table 5. The charge transfer of the corrosion process and the double layer behaviour were simulated by the equivalent circuit consisting of an electrolyte resistance *R*_S_ in series with the parallel combination of the double-layer capacitance (*C_dl_*) and the charge transfer resistance (*R_ct_*) [37,39]. Due to the imperfection of the metal surface, the double layer capacitance (*C_dl_*) is simulated using a constant phase element (*CPE*) [14]. The impedance of *CPE* is described by Equation (10):

(10) $$Z_{CPE}=\frac{1}{Q{\left(j\omega \right)}^{n}}$$

where *Q* stands for *CPE* constant, *n* is the exponent, *j* is the imaginary number, and *ω* is the angular frequency at which *Z* reaches its maximum value. The parameter *n* quantifies the imperfections of the surface. For *n* = 1, *CPE* is a pure capacitor, and for *n* = −1, *CPE* is an inductor. The values of the double layer capacity were calculated from the following Equation (11) [20]:

(11) $$C_{dl}=\frac{1}{\omega R_{ct}}=\frac{1}{2\pi f_{max}R_{ct}}$$

where *f_max_* represents the frequency at which *Z* reaches its maximum value on the Nyquist plot.

**Figure 5:**
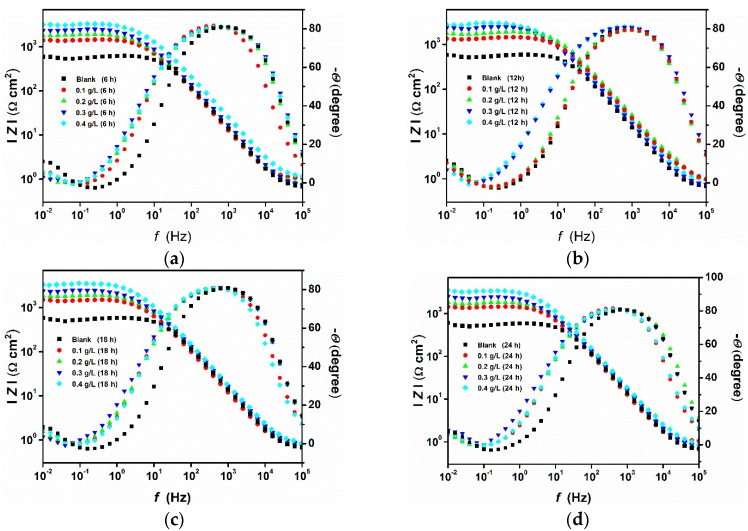
Bode Modulus and Bode Phase plots for pure aluminium in 1-M HCl in the absence and presence of different concentrations of GG after 6 (**a**), 12 (**b**), 18 (**c**), and 24 (**d**) h of immersion.

Inspection of Table 5 reveals that the charge transfer resistance increases with an increase in inhibitor concentration, going from 624 Ω cm^2^ up to 3166 Ω cm^2^ after 6 h of immersion for the blank and inhibited solution (i.e., 0.4 g/L), respectively. By contrast, it follows from the data that the double-layer capacitance values vary in inverse proportions to that observed for the charge transfer resistance, going from 16.15 µF cm^−2^ to 12.73 µF cm^−2^ for the blank and inhibited solution, respectively. *R_ct_* is associated with the corrosion resistance of the process; therefore, a large charge transfer resistance is suggestive of a slower corrosion process, which in turn indicates better inhibition performance due to the adsorption of the inhibitor on the electrode surface. On the other hand, a decrease in *C_dl_* of the metal compared to the blank solution with increase in inhibitor concentration points to the formation of a growing protective layer on the metal surface [33,34,40], in accordance with the Helmholtz model given by Equation (12) [33,34,41].

(12) $$C_{dl}=\frac{\varepsilon \varepsilon_{0}A}{d}$$

where *ε* is the dielectric constant of the medium, ε_0_ is the vacuum permittivity, *A* is the electrode area, and *δ* is the thickness of the protective layer. The Bode modulus (Figure 5) agrees with the Nyquist spectra in which it can be seen that the impedance modulus was observed to increase with increasing GG concentration, which indicates reduced corrosion rates in the inhibited solutions. Moreover, the effect of the inhibitor can also be seen from the Bode phase angle plots, where the phase angle becomes broader after the addition of the inhibitor. The broadening is likely due to the formation of a porous inhibitor film on the electrode surface.

The inhibition efficiency values were calculated from the *R_ct_* values and are given in Table 5. It follows from the data that the inhibition efficiency increases with increasing concentrations of GG, going from 56.73% to 80.29% after 6 hours of immersion at 0.1 g/L and 0.4 g/L of GG, respectively, which indicates that GG exhibits good inhibitive performance for pure aluminium in a 1-M HCl solution. Moreover, it is also apparent from the data that the immersion time causes little changes in the performance of the inhibitor, with the maximum inhibition efficiency obtained at 25 °C found to be 82.83% at 0.4 g/L after 24 h of immersion. These results suggest a rapid formation of a protective layer on the metal surface in the first six hours of the experiment, after which the active sites on the surface are almost occupied by the inhibitor and, therefore, the corrosion process becomes stable. Peter et al. also reported a similar result [16].

### 3.3. Surface Analysis

#### 3.3.1. FTIR and Raman/SERS Analysis

**Figure 6:**
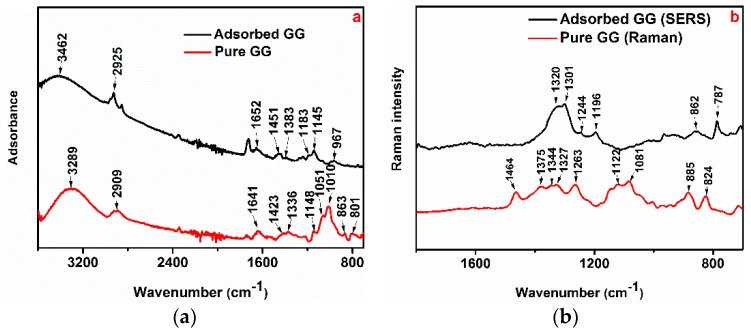
FTIR (**a**) and Raman (**b**) spectra of native Guar gum and surface-adsorbed Guar gum in the presence of the optimum concentration of GG (i.e., 0.4 g/L).

Vibrational spectroscopy such as infrared and Raman/SERS spectroscopies can be useful techniques to identify the functional groups on the polymer chain of polysaccharides, (e.g., O-H, C-H, etc.) and their interaction with the metal surface based on their specific frequencies. The FTIR and Raman spectra of the pure GG and the adsorbed GG on the metal surface after 24 h of immersion are shown in Figure 6. The spectra are stack plotted to facilitate the comparison of any similarities or differences which might occur during the adsorption of GG on the aluminium surface. Any differences between the shape of the two spectra (e.g., changes in frequency or intensity) can be used to gain useful information regarding the adsorption mechanism of the tested inhibitor on the metallic surface. The spectrum of the pure GG (Figure 6a) shows a peak at 3289 cm^−^^1^ which is ascribed to the extensive intramolecular hydrogen bonds and stretching vibration of the -OH groups [12,14,17,18]. The peak at 2909 cm^−^^1^ for the pure GG is assigned to the antisymmetric and symmetric C-H stretching vibrations of the methylene hydroxyl groups (-CH_2_OH) [12,14,17,18]. The spectrum of the aluminium surface adsorbed GG sample shows narrowed and shifted peaks around the same frequency (Figure 6a). Some authors [6,14,17] reported that the peaks changing phenomenon may be due to the binding of some of the hydroxyl groups of the GG inhibitor with the metal surface via H-bond formation. 

The region between 1200 and 950 cm^−1^ is the so-called fingerprint region of polysaccharides, where the position and intensity of the bands are specific for every polysaccharide. Briefly, the peaks in the 1500–1200 cm^−1^ region are associated with the C-H bending/scissoring, twisting, and rocking vibrations while the bands at 1200–1000 cm^−1^ and 800–900 cm^−1^ are due to the symmetrical and asymmetrical ring breathing vibrations of C-C-O, C-OH, and C-O-C associated with the glucoside (1–4) linkage of galactose and (1–6) linkage of mannose [12,14,17,18]. The shift of the peaks between these regions may be attributed to a possible interaction between the endocyclic oxygen atom of mannose unit of GG and C(6)-O-C(1) and/or C(1)-O-C(4) of the same monosaccharide unit with the surface [14,17,18]. 

In order to further investigate this region, Raman and SERS analysis of the native GG and the adsorbed GG were respectively performed and are shown in Figure 6b. The Raman in the region of 850–950 cm^−^^1^ correspond to the skeleton mode of the anomeric skeletal configuration (α or β conformers) and glycosidic linkages [42]. It can be seen from the figure that the peaks in the Raman spectrum of the native GG are blue shifted in comparison to the SERS spectrum of the adsorbed GG. This shift is likely due to the formation of the metal-polysaccharide complex as mentioned above. The involvement of C(6) oxygen of mannose unit in chelate formation is further supported by the blue shift at the 1300 cm^−1^ band, attributed to C-H wagging and bending of either -CH or -CH_2_ [42]. 

The overall features of the FTIR and Raman spectra shown in Figure 6 suggested that there is a strong adsorption of GG on the surface of the metal. This conclusion was deduced from the reduction of the intensities and/or shift of the position of all major characteristic peaks of the adsorbed polysaccharide. These results are in agreement with the previous works reported regarding the adsorption of Gum inhibitor on steel surface [6,14,17,18].

#### 3.3.2. SEM Morphology

**Figure 7:**
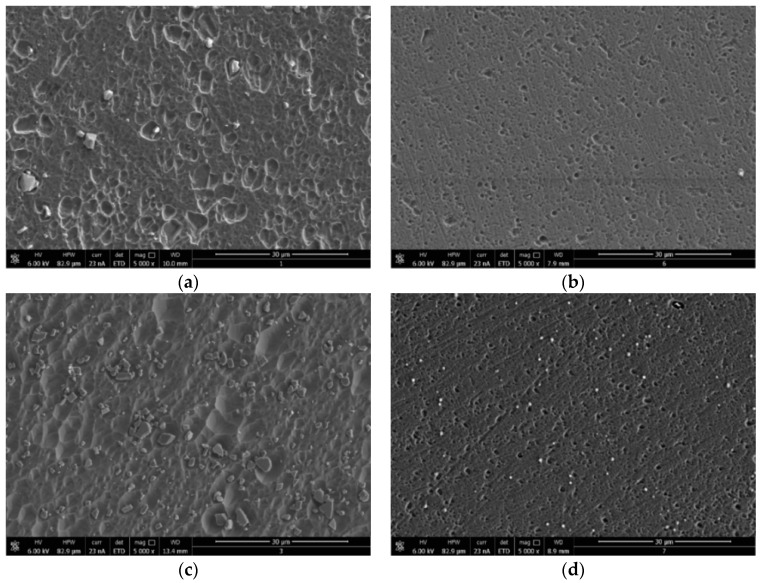
SEM images of the aluminium surface after 24 h of immersion in 1-M HCl without and with the presence of the optimum concentration of GG (i.e., 0.4 g/L) (**a**) in the blank solution and (**b**) in the presence of GG at 25 °C, respectively, and (**c**) in the blank solution and (**d**) in the presence of GG at 45 °C, respectively.

Figure 7 shows the SEM images of the surface of specimens after immersion in the tested solution without and with the addition of the optimum concentration of GG (i.e., 0.4 g/L) at 25 and 45 °C after 24 h of immersion. It can be seen from Figure 7a that, at 25 °C, the surface is badly corroded as a result of the local breakdown of the oxide layer distributed all over the surface. The size and depth of the pits increased by increasing the temperature (Figure 7c). In contrast, the damage of aluminium surface diminished in the presence of inhibitor. It can be seen that the size and depth of the pits are reduced and that the sample surface shows a much smoother surface both at 25 and 45 °C (Figure 7b,c). Abdallah also reported a similar result [13]. The author reported that GG increased the resistance to pitting attack in the presence of chloride ions. Table 6 shows the EDS analysis of the corroded sample in the blank and inhibited solutions. The data shows the presence of aluminium and oxygen, suggesting the presence of aluminium oxide/hydroxide in both inhibited and uninhibited solution. However, it follows from the table that, after the addition of GG, the amount of oxygen on the metal surface increased and that there is the presence of a high percentage of carbon element. It should be noted that carbon and oxygen are part of the chemical composition of the inhibitor and that the appearance of C and the enhancement in O upon introducing GG to the corrosive medium are likely due to the adsorption of GG on the surface of the metal. Moreover, it follows from the data that the percentage of Al decreased in the presence of GG. This is likely to occur due to the overlying effect of the inhibitor film.

### 3.4. Mechanism of Inhibition

Guar gum is a polysaccharide consisting of a backbone of D-mannopyranose monomer units attached to each other by β-(1→4) linkage with a side-branch consisting of a single α-D-galactopyranose linked to the mannose unit by α-(1→6) linkages (Figure 1). It is believed that the corrosion protection ability of GG is achieved via adsorption of its molecules on the aluminium surface through some adsorptive centres present in the inhibitor structure such as heteroatoms (i.e., oxygen from the heterocyclic moiety) and/or functional groups (i.e., hydroxyl functional group) [13,14,17,18]. From the values of Δ*G_ads_* presented in Table 3, it can be deduced that the adsorption process of GG on the metal surface occurs via a mixed-type interaction (i.e., physical and chemical adsorption) [14,17,18]. Therefore, four types of adsorption or a combination of them may take place in the inhibiting phenomena involving GG on the pure aluminium surface in a 1-M HCl solution.

#### 3.4.1. Electrostatic Interaction

In acid solution, the hydroxyl functional groups present on the GG macromolecules can be protonated such that the polymer exists as a polycation, existing in equilibrium with the corresponding molecular form, according to Equation (13):

(13) $$\mathrm{GG}+{\mathrm{xH}}^{+}↔{\left[{\mathrm{GGH}}_{x}\right]}_{\left(\mathrm{sol}\right)}^{x+}$$

where [GGH_x_]^x+^_(sol)_ is the protonated inhibitor in solution. Moreover, it is well known that aluminium and its alloys form a passive layer on their surface [34]. In acid media, the oxide or hydroxide surface can be protonated and, therefore, generates positively charged surfaces, according to Equation (14) [34]:

(14) $$\mathrm{AlOH}+H^{+}↔{\mathrm{AlOH}}_{2}^{+}$$

**Figure 8:**
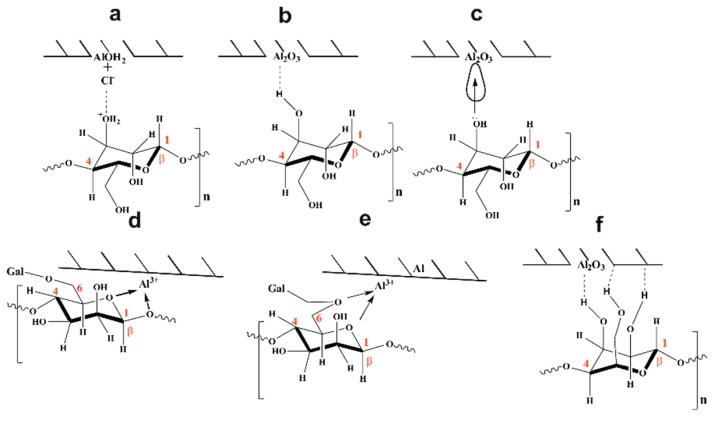
Schematic representation of possible adsorption models of GG on the aluminium surface in an acid medium: (**a**) electrostatic interaction with Cl^−^ ions; (**b**) H-bond formation; (**c**) coordinate bond with the metal; (**d**,**e**) chelating action; and (**f**) horizontal orientation.

The accumulation of ${\mathrm{AlOH}}_{2}^{+}$ species accounts for the positive surface charge [34,43,44]. This excess positive charge will electrostatically attract any chloride ions present in solution, changing the surface charge from positive to negative [20,34]. The adsorption of GG on the aluminium surface may occur through electrostatic interaction between the adsorbed Cl^−^ ions and the partially positive charged GG macromolecules (Figure 8a), according to Equations (15) and (16) [45].

(15) $${\mathrm{Cl}}_{\left(\mathrm{sol}\right)}^{-}\to {\mathrm{Cl}}_{\left(\mathrm{ads}\right)}^{-}$$

(16) $${\mathrm{Cl}}_{\left(\mathrm{ads}\right)}^{-}+{\left[{\mathrm{GGH}}_{x}\right]}_{\left(\mathrm{sol}\right)}^{x+}↔{\left({\mathrm{Cl}}^{-}-{\left[{\mathrm{GGH}}_{x}\right]}^{x+}\right)}_{\left(\mathrm{ads}\right)}$$

where Cl^−^_(sol)_ and [GGH_x_]^x+^_(sol)_ are the chloride ion and the protonated inhibitor, respectively, in the bulk of the solution while Cl^−^_(ads)_ refers to the adsorbed chloride ions on the metal surface and where (Cl^−^−[GGH_x_]^x+^)_(ads)_ represents the electrostatic interaction between the adsorbed anions and the pronated inhibitor.

#### 3.4.2. Adsorption via H-Bond Formation

In acid solution, the cathodic and anodic reactions are due to the reduction of hydrogen and to the dissolution of the metal, respectively [46,47]. It can be seen from the polarisation curves (Figure 2) that the cathodic current densities are more greatly affected after the addition of GG than the anodic ones. Roy et al. [17] and Messali [14] et al. presented some FTIR analyses regarding the adsorption of GG on the steel surface in acid media. The authors reported that the characteristic broad peak at circa 3300 cm^−1^, attributed to intramolecular hydrogen bonding and hydroxyl groups stretching vibration in GG structure, shifted and/or narrowed down after its adsorption on the metal surface. The authors suggested that this change in shape might be attributed to the formation of hydrogen bonds between the hydroxyl groups of the inhibitor structure with H^+^ adsorbed on the steel surface. FTIR measurements reported in this study seem to support such a hypothesis. Figure 6 shows that the intensity of the characteristic peaks associated with the hydroxyl groups shifted after the adsorption of GG on the metal surface. This behaviour may be attributed to the formation of hydrogen bonds established between the oxidized aluminium surface and the hydroxyl groups of the inhibitor as displayed in Figure 8b. 

#### 3.4.3. Chemical Adsorption

As mentioned before, the protonated molecules of GG are in equilibrium with the neutral molecules according to Equation (13) [14,17,41]. These neutral molecules can also be adsorbed via a coordinated type of bonds between the lone pair of the electrons of the heteroatoms (e.g., oxygen) and the empty p-orbital of Al atom on the metal surface (Figure 8c) [4,28,44]. However, as suggested by Khaled and coauthor, in the presence of an oxide layer, like in the case of aluminium in an aqueous solution, the direct coordination of oxygen to an exposed metal atom seems a remote event [34]. 

#### 3.4.4. Chelate Formation

The adsorption of GG via chelate complex on the anodic sites on metal was first suggested by Abdallah in a study regarding the adsorption of GG on carbon steel surface in sulphuric media [13]. The author suggested that the carbohydrate-metal complex may be formed through a coordinated type of bond between the endocyclic oxygen atom of the mannose backbone units, together with the oxygen atom from the glycosidic C(1)-O-C(4) linkage, with freshly generated Fe^2+^ ions present on anodic sites ions (Figure 8d). Roy et al. [17] and Messali et al. [14] through FTIR analyses confirmed the formation of this carbohydrate-metal complex; however, the authors observed that the complex may be formed by the endocyclic oxygen atom and the oxygen atom from C(6)-O-C(1) linkage rather than the C(1)-O-C(4) linkage (Figure 8e). In both cases, the complexes may adsorb on the steel surface through van der Waals forces to form a protective film to keep the metal surface from corrosion [21]. FTIR and Raman/SERS measurements reported in this study also seem to support such a hypothesis. However, a closer inspection of the polarisation curves presented in Figure 2 reveals that the cathodic reaction is much more greatly suppressed compared to the anodic reaction. This suggests that the adsorption of GG via H-bond formation may account for the most effective inhibition action.

#### 3.4.5. Mode of Adsorption (Horizontal Orientation)

From Table 1, it can be deduced that the extent of GG protection depends upon its concentration. The increase in *IE*% may be attributed to the formation of a protective layer as a result of the adsorption of GG on the aluminium surface. It is well known that corrosion inhibitors generally display high inhibitor efficiency at low concentrations. In this study, the high values of *IE*% observed for the tested inhibitor, even at low concentrations (i.e., 48.85% at 0.1 g/L at 25 °C), could be explained on the basis of the flat adsorption of the GG molecules on the metallic surface [38]. Abdallah [4] first hypothesised that the formation of the carbohydrate-metal complex forced the Guar gum molecules to lie horizontally on the metal surface. In this case, the inhibitor molecules, lying approximately parallel to the surface, would occupy a larger surface area, thus increasing the surface coverage and, consequently [48], the corrosion efficiency. However, as stated before, the cathodic reaction is much more greatly suppressed compared to the anodic reaction. Therefore, is more likely that the high inhibitor efficiency displayed at low concentrations by GG may be due to the simultaneous adsorption of the -OH groups on the cathodic side of the metal that forced the mannose units and, thus, the molecules of the inhibitor to lie parallel to the surface (Figure 8f).

## 4. Conclusions

Considering the obtained results, the inhibition effect of Guar gum (GG) on pure aluminium in a 1-M HCl solution can be summarised in the following points:

- GG was found to be a good corrosion inhibitor for pure aluminium in the tested solution.
- The inhibition efficiency increases with an increase in inhibitor concentration but decreases as the temperature increases. The maximum inhibition efficiencies were 82.85 and 64.30 at 25 °C and 45 °C, respectively.
- Time has little effect on the corrosion inhibition efficiency of the tested inhibitor, suggesting that GG is able to protect the metal for long exposure periods.
- The inhibition action of Guar gum is attributed to its horizontal adsorption on the metallic surface.
- The adsorption of GG on the aluminium surface follows the Temkin adsorption isotherm model.
- The negative values of the free energy of adsorption indicate spontaneous adsorption of GG on the aluminium surface. Moreover, the values of Δ*G_ads_* indicate that the adsorption process is a mixed-type interaction (i.e., physical and chemical adsorption). The values of *E_a_* and *Q_ads_* further support the mixed-type adsorption.
- The potentiostatic polarisation measurements indicate that GG acts as a cathodic inhibitor.
- FTIR and Raman spectroscopies suggested that the most probable inhibition action of GG is due to its adsorption of the metal surface via H-bond formation.

## Figures and Tables

**Table 1:** Corrosion rate and inhibition efficiency obtained from weight loss measurements for pure aluminium at various concentrations of GG after 24 h of immersion at 25 and 45 °C.

*C_inh_* (g L^−1^)	Corrosion Rate (mg cm^−2^ d^−1^)		*IE* (%)	
-	25 °C	45 °C	25 °C	45 °C
Blank	5.65	18.50	-	-
0.1	2.89	13.87	48.85	25.03
0.2	1.91	11.07	66.19	40.16
0.3	1.35	9.05	76.11	51.08
0.4	0.95	7.37	83.19	60.16

**Table 2:** Potentiodynamic polarisation parameters for pure aluminium without and with different concentrations of GG at 25 and 45 °C after 24 h of immersion.

*C*_inh_ (g L^−1^)	*β*_a_ (V dec^−1^)	*β*_c_ (V dec^−1^)	*I*_corr_ (µA cm^−2^)	*E*_corr_ (V/SCE)	*IE* (%)
25 °C					
Blank	0.294	0.280	8.98	−0.967	-
0.1	0.251	0.212	3.96	−0.996	55.90
0.4	0.215	0.220	1.42	−1.049	84.19
45 °C					
Blank	0.293	0.185	29.9	−1.03	-
0.1	0.161	0.137	22.4	−1.04	25.08
0.4	0.164	0.124	14.4	−1.07	51.84

**Table 3:** Parameters of the linear regression from Langmuir and Temkin adsorption isotherms at 25 and 45 °C.

Temperature (°C)	R^2^	Intercept	Slope	*a*	*K*	Δ*G_ads_* (kJ mol^−1^)
Langmuir						
25	0.999	0.115	0.920	-	8.70	−22.48
45	0.996	0.316	0.885	-	3.16	−21.32
Temkin						
25	0.999	1.059	0.570	−2.02	72.09	−27.73
45	0.989	0.819	0.577	−2.00	26.27	−26.92

**Table 4:** Apparent activation energies (*E_a_*) and heat of adsorption (*Q_ads_*) calculated in the temperature range of 25–45 °C.

*C_inh_* (g L^−1^)	*E_a_* (kJ mol^−1^)	*Q_ads_* (kJ mol^−1^)
Blank	46.78	-
0.1	61.86	−41.45
0.2	69.30	−42.22
0.3	75.04	−43.99
0.4	80.80	−46.81

**Table 5:** Electrochemical impedance parameters for pure aluminium in a 1-M HCl solution with and without the presence of various concentrations of GG at 25 °C after 6, 12, 18, and 24 h of immersion.

Time (h)	*R_s_* (Ω cm^2^)	*Q* (μΩ^−1^ s^n^ cm^−2^)	*n*	*R* (Ω cm^2^)	*C_dl_* (μF cm^−2^)	*IE* (%)
Blank						
6	0.63	19.8	0.94	624	16.15	-
12	0.63	20.2	0.94	589	17.11	-
18	0.63	20.7	0.93	585	17.23	-
24	0.64	20.6	0.93	589	17.11	-
0.1 g L^−1^						
6	0.63	23.5	0.93	1442	11.85	56.73
12	0.63	19.4	0.92	1429	11.96	58.78
18	0.76	23.8	0.93	1481	11.54	60.50
24	0.76	23.5	0.93	1455	11.74	59.52
0.2 g L^−1^						
6	0.76	18.8	0.93	1836	13.72	66.01
12	0.70	16.9	0.92	1893	13.31	68.89
18	0.78	17.9	0.93	1910	13.19	69.37
24	0.66	20.5	0.92	1799	14.01	67.26
0.3 g L^−1^						
6	0.65	22.0	0.91	2498	10.09	75.02
12	0.66	28.2	0.90	2582	9.76	77.19
18	0.77	23.6	0.91	2480	10.16	76.41
24	0.77	17.5	0.93	2490	10.12	76.35
0.4 g L^−1^						
6	0.65	14.2	0.91	3166	12.73	80.29
12	0.76	19.5	0.92	3116	12.94	81.10
18	1.05	22.5	0.92	3338	12.08	82.47
24	1.03	23.3	0.92	3431	11.75	82.83

**Table 6:** EDS analysis result of the aluminium surface after 24 h of immersion in 1-M HCl without and with the presence of the optimal concentration of GG (i.e., 0.4 g/L).

Element	Weight %			
-	25 °C		45 °C	
-	Blank	GG	Blank	GG
C	-	5.7	-	4.9
O	1.6	2.9	1.2	2.6
Si	1.1	1.3	0.9	1.1
Cl	0.4	0.2	0.5	0.2
Al	96.9	89.9	97.4	91.2

## Supplementary Materials

The following are available online at https://www.mdpi.com/1996-1944/12/16/2620/s1, Table S1: Comparison of reported inhibition efficiency of some other corrosion inhibitors originated from natural products with the present inhibitor.
