# Supplementary material for: Guar Gum as an Eco-Friendly Corrosion Inhibitor for Pure Aluminium in 1-M HCl Solution
Source: Materials (Basel). 2019 Aug 16;12(16):2620. doi: 10.3390/ma12162620 (PMC6720888; doi:10.3390/ma12162620)
Supplement: Supplementary file 1 [file materials-12-02620-s001.pdf]

materials

Article

Guar Gum as an Eco-Friendly Corrosion Inhibitor for Pure Aluminium in 1-M HCl Solution

Gaetano Palumbo [1,*], Katarzyna Berent [2], Edyta Proniewicz [1] and Jacek Banaś [1]

Table S1. Comparison of reported inhibition efficiency of some other corrosion inhibitors originated from natural products with the present inhibitor.

Inhibitors derived from natural products	Metal Substrate	Corrosive Media	Inhibitor conc. at which maximum inhibition efficiency is observed	Temp. (°C)	Inhibition efficiency (%)	Time of exposure (h)	Reference
Guar Gum	Carbon Steel	1M H_2SO_4 containing NaCl	1500 ppm	25	93.88	24	[13]
Gum Arabic	API 5L X42 steel	1 M HCl	2000 ppm	25	92	1	[20]
Gum Arabic	Mild Steel	0.1 M H_2SO_4	1500 ppm	30	83.09	6	[7]
Gum Arabic	Mild Steel	0.1 M H_2SO_4	500 ppm	30	21.9	168	[19]
Gum Arabic	Aluminium	0.1 M NaOH	500 ppm	30	50.24	-	[9]
Polysaccharide from Plantago	Carbon Steel	1 M HCl	1000 ppm	30	89.1	6	[11]
Polyacrylamide grafted Guar gum	Mild Steel	1 M HCl	500 ppm	30	93<br>≈ 70	5<br>168	[17]
Gum Arabic	Mild Steel	0.1 M H_2SO_4	500ppm	30	21.84	168	[5]
Gum Arabic	Aluminium	0.1 M H_2SO_4	500ppm	30	79.65	168	[5]
gum from Raphia hookeri	Mild Steel	0.1 M H_2SO_4	500 ppm	30	≈ 38<br>71.9	24<br>168	[22]
Guar Gum	Carbon Steel	2.0 M H_3PO_4	1000 ppm	25	92.8	6	[14]
Xanthan gum	Carbon Steel	1 M HCl	1000ppm	30	74.24	6	[10]
Gum Acacia	Mild Steel	0.5 M HCl<br>0.5 M H_2SO_4	600 ppm	25	44.74<br>16.98	168	[6]
Guar Gum	Aluminium	1 M HCl)	800 ppm	25<br>45	83.19<br>60.16	24	[13]
